# Krempfielins Q and R, Two New Eunicellin-Based Diterpenoids from the Soft Coral *Cladiella krempfi*

**DOI:** 10.3390/ijms151221865

**Published:** 2014-11-27

**Authors:** Chi-Jen Tai, Uvarani Chokkalingam, Yang Cheng, Shou-Ping Shih, Mei-Chin Lu, Jui-Hsin Su, Tsong-Long Hwang, Jyh-Horng Sheu

**Affiliations:** 1 Department of Marine Biotechnology and Resources, National Sun Yat-sen University, Kaohsiung 804, Taiwan; E-Mails: jean801023@hotmail.com (C.-J.T.); uvaranichem@gmail.com (U.C.); jack1991106@yahoo.com.tw (Y.C.); 2 Doctoral Degree Program in Marine Biotechnology, National Sun Yat-sen University and Academia Sinica, Kaohsiung 804, Taiwan; 3 Graduate Institute of Marine Biotechnology, National Dong Hwa University, Pingtung 944, Taiwan; E-Mails: m6430005@hotmail.com (S.-P.S.); jinx6609@nmmba.gov.tw (M.-C.L.); x2219@nmmba.gov.tw (J.-H.S.); 4 National Museum of Marine Biology & Aquarium, Pingtung 944, Taiwan; 5 Graduate Institute of Natural Products, Chang Gung University, Taoyuan 333, Taiwan; E-Mail: htl@mail.cgu.edu.tw; 6 Department of Medical Research, China Medical University Hospital, China Medical University, Taichung 404, Taiwan; 7 Graduate Institute of Natural Products, Kaohsiung Medical University, Kaohsiung 807, Taiwan

**Keywords:** *Cladiella krempfi*, eunicellin-based diterpenoid, anti-inflammatory activity

## Abstract

Two new eunicellin-based diterpenoids, krempfielins Q and R (**1** and **2**), and one known compound cladieunicellin K (**3**) have been isolated from a Formosan soft coral *Cladiella krempfi*. The structures of these two new metabolites were elucidated by extensive spectroscopic analysis. Anti-inflammatory activity of new metabolites to inhibit the superoxide anion generation and elastase release in *N*-formyl-methionyl-leucyl phenylalanine/cytochalasin B (FMLP/CB)-induced human neutrophil cells and cytotoxicity of both new compounds toward five cancer cell lines were reported.

## 1. Introduction

**Chart 1:**
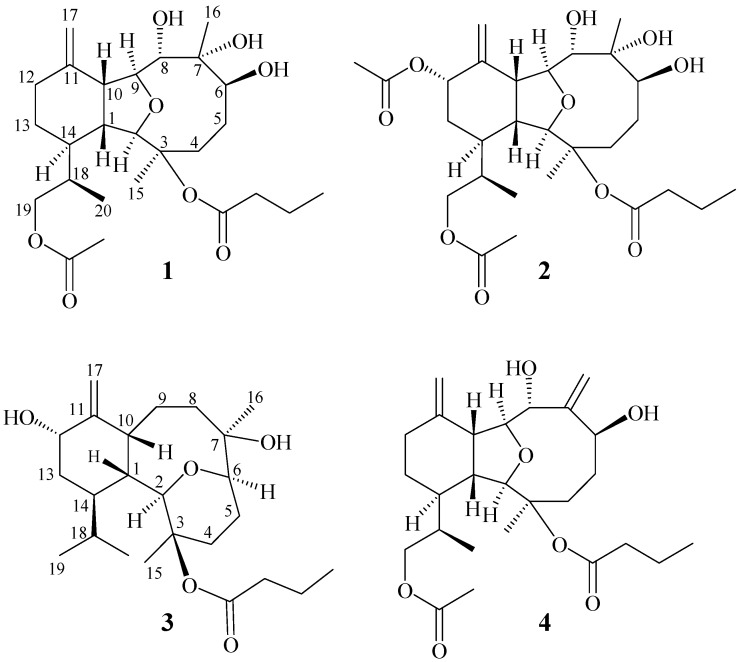
Structures of metabolites **1–4**.

Soft corals of the genus *Cladiella* have been known to be rich sources of eunicellin-type metabolites and several bioactivities of these compounds have been studied [1,2,3,4,5,6,7,8,9,10,11,12,13,14,15,16,17,18]. Our previous studies on the soft coral *Cladiella krempfi* have resulted in the isolation of a series of new eunicellin-based diterpenoids, krempfielins A–P [15,16,17,18]. Our contineous investigation on the chemical constituents of soft coral *C.* *krempfi* has afforded two new compounds, krempfielins Q and R (**1** and **2** in Chart 1), and one minor known compound cladieunicellin K (**3**) [11]. The molecular structures of **1** and **2**, including the relative configurations, were established by the detailed spectroscopic analysis and by comparison with related physical and spectral data of known compound, krempfielin E (**4**) [16]. For the anti-inflammatory activity of these two new compounds to inhibit the superoxide anion generation and elastase release in *N*-formyl-methionyl-leucyl phenylalanine/cytochalasin B (FMLP/CB)-induced human neutrophils and the cytotoxicity of them against five human cell lines, human T cell lymphoblast-like cell line (CCRF-CEM), human erythromyeloblastoid leukemia (K562), human acute lymphoblastic leukemia cell line (Molt 4), human ductal breast epithelial tumor cell line (T47D) and human colorectal adenocarcinoma cell line (DLD-1) were also evaluated.

## 2. Results and Discussion

**Figure 1:**
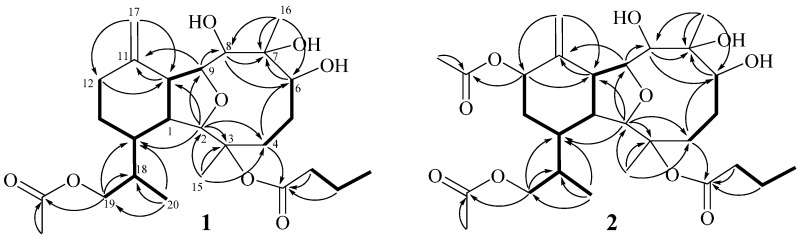
Selected COSY (▬) and HMBC (→) correlations of **1** and **2**.

Krempfielin Q (**1**) showed the molecular ion peak [M + Na]^+^ at *m*/*z* 505.2779 in the HRESIMS (Figure S1) and established a molecular formula of C_26_H_4__2_O_8_, implying six degrees of unsaturation. The IR absorption bands at ν*_max_* 3445 and 1733 cm^−^^1^ revealed the presence of hydroxy and ester carbonyl functionalities, respectively. Its ^13^C NMR spectrum (Figure S2) showed signals of 26 carbons (Table 1) which were assigned by the assistance of the distortionless enhancement by polarization transfer (DEPT) spectrum to five methyls (including one acetate methyl δ_C_ 21.1), seven sp^3^ methylenes, one sp^2^ methylene, eight sp^3^ methines (including four oxymethines), two sp^3^ and three sp^2^ quaternary carbons (including two ester carbonyls). The NMR spectroscopic data of **1** (Figures S2,S3 and Table 1) displayed signals for 1,1-disubstituted double bond (δ_C_ 148.4 C, 110.7 CH_2_; δ_H_ 4.91 and 4.81 s). Two ester carbonyls (δ_C_ 172.2 and 171.2) were assigned from the ^13^C NMR spectrum and their signals were correlated with the methylene protons (δ_H_ 2.33, 2H, m) of an *n*-butyrate and protons of an acetate methyl (δ_H_ 2.08 s, 3H), respectively. Therefore, the remaining three degrees of unsaturation identified **1** as a tricyclic molecule. The ^1^H-^1^H correlation spectroscopy (COSY) and heteronuclear multiple bond correlation (HMBC) correlations (Figure 1) were further used for establishing the molecular skeleton of **1**. It was found that the COSY experiment showed the presence of three isolated consecutive proton spin systems. These evidences and the analysis of HMBC spectrum (Figure 1) suggested that **1** is an eunicellin-based diterpenoid. Furthermore, the acetoxy group attaching at C-19 was confirmed by the HMBC correlations from oxymethylene (δ_H_ 3.96 (H_2_-19)) and acetate methyl protons (δ_H_ 2.08) to the ester carbonyl carbon appearing at δ 171.2 (C). Thus, the remaining *n*-butyryloxy group showed to be positioned at C-3, which was confirmed by an oxygen-bearing quaternary carbon resonating at δ 86.0 ppm. On the basis of above analysis, the planar structure of **1** was established.

**Figure 2:**
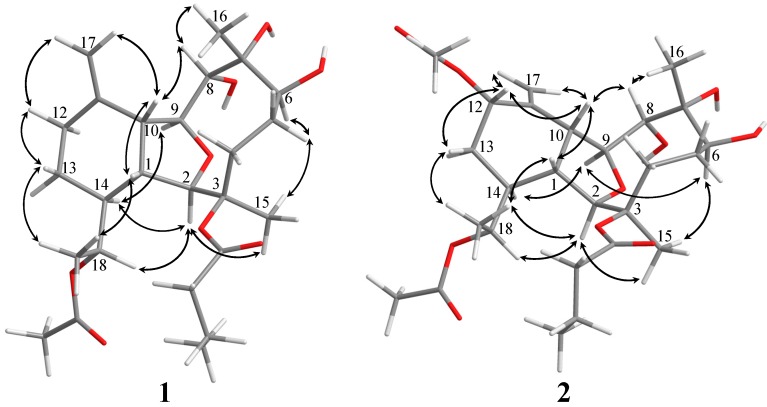
Key NOESY (↔) correlations for **1** and **2**.

The relative configuration of **1** was deduced by the analysis of nuclear Overhauser effect (NOE) correlations, as shown in Figure 2. The observation of the NOE correlations of H-1 with H-10 and H_3_-20 suggested that these protons had the same orientation and were assumed to be β-oriented. The NOE interactions found between the oxymethine proton H-8 with H-10 and H_3_-16 assigned the α-orientation of the two hydroxy groups positioned at C-7 and C-8. The NOE correlations of H-2 with both H-14 and H_3_-15, but not with H-1 and H-10; H-14 with both H-9 and H_2_-19; and H-5α (δ_H_ 1.62) with both H-6 and H_3_-15, permitted that H-2, H-6, H-9, H-14, and H_3_-15 were assigned to be α-oriented. Furthermore, the configuration of C-18 was suggested to be *R* * on the basis of NOE correlations of H-1 with H_3_-20, H-14 with H_2_-19, and H-2 with H-18. The relative configuration of **1** was thus established. Comparison of the ^1^H and ^13^C NMR spectroscopic data of **1** with those of its 7,16-dehydration derivative, krempfielin E (**4**) [16], further confirmed the structure of **1**.

**Table 1:** ^13^C and ^1^H NMR data for compounds **1–2**.

C	1 ^a^		2 ^b^	
	δ_C_	δ_H_	δ_C_	δ_H_
**1**	44.9 (CH) ^c^	2.27 m ^d^	43.5 (CH)	2.35 m
**2**	92.3 (CH)	3.54 br s	90.8 (CH) ^e^	3.63 d (1.5)
**3**	86.0 (qC)		86.0 (qC)	
**4**	35.6 (CH_2_)	1.84 dd (14.4, 10.4) ^f^	34.6 (CH_2_) ^e^	1.88 m
		2.61 dd (14.4, 9.2)		2.49 dd (14.0, 9.0)
**5**	29.5 (CH_2_)	1.42 m	29.9 (CH_2_)	1.43 m
		1.62 m		1.70 m
**6**	77.6 (CH)	4.64 d (6.0)	77.0 (CH)	4.63 d (7.5)
**7**	79.6 (qC)		79.4 (qC)	
**8**	80.0 (CH)	3.60 br d (8.4)	79.1 (CH)	3.50 br t (8.0)
**9**	81.3 (CH)	3.85 dd (9.2, 6.4)	82.2 (CH)	4.05 dd (9.5, 5.5)
**10**	53.3 (CH)	3.34 t (6.8)	50.6 (CH)	3.35 t (6.5)
**11**	148.4 (qC)		143.0 (qC)	
**12**	31.4 (CH_2_)	2.08 m	72.8 (CH)	5.44 d (4.5)
		2.31 m		
**13**	25.4 (CH_2_)	1.17 dd (12.8, 2.8)	29.4 (CH_2_)	1.50 m
		1.68 m		1.88 m
**14**	38.9 (CH)	1.51 m	32.7 (CH)	1.90 m
**15**	23.1 (CH_3_)	1.41 s	23.2 (CH_3_)	1.46 s
**16**	17.7 (CH_3_)	1.26 s	17.7 (CH_3_)	1.26 s
**17**	110.7 (CH_2_)	4.81 s	116.3 (CH_2_) ^e^	5.23 s
		4.91 s		
**18**	34.1 (CH)	1.92 m	34.0 (CH)	1.99 m
**19**	67.7 (CH_2_)	3.96 d (7.2)	67.8 (CH_2_)	3.90 dd (11.0, 7.0)
				4.02 dd (11.0, 7.0)
**20**	11.0 (CH_3_)	0.85 d (6.4)	11.4 (CH_3_)	0.90 d (6.5)
**3-OCOPr**	172.2 (qC)		172.2 (qC)	
	37.3 (CH_2_)	2.33 m	37.3 (CH_2_)	2.30 m
	18.4 (CH_2_)	1.68 m	18.3 (CH_2_)	1.63 m
	13.7 (CH_3_)	0.99 t (7.2)	13.6 (CH_3_)	0.98 t (7.0)
**12-OAc**			170.4 (qC)	
			21.5 (CH_3_)	2.08 s
**19-OAc**	171.2 (qC)		171.0 (qC)	
	21.1 (CH_3_)	2.08 s	20.9 (CH_3_)	2.07 s

^a^ ^13^C and ^1^H spectra recorded at 100 and 400 MHz in CDCl_3_; ^b^ ^13^C and ^1^H spectra recorded at 125 and 500 MHz in CDCl_3_; ^c^ Deduced from DEPT; ^d^ Mutiplicity m deduced from HSQC; ^e^ Broad signal; and ^f^*J* values (Hz) in parentheses.

Krempfielin Q (**2**) showed the molecular ion peak [M + Na]^+^ at *m*/*z* 563.2835 in the HRESIMS (Figure S4) which established a molecular formFfigure sula of C_2__8_H_44_O_10_, implying seven degrees of unsaturation for this compound. The IR absorptions at ν*_max_* 3444 and 1732 cm^−^^1^ were consistent with the presence of hydroxy and ester carbonyl functionalities. The ^13^C NMR spectrum of **2** showed signals of 28 carbons (Figure S5 and Table 1), which were differentiated by the DEPT spectrum as six methyls (including two acetate methyls δ_C_ 21.5 and 20.9), six sp^3^ methylenes, one sp^2^ methylene, nine sp^3^ methines (including five oxymethines), two sp^3^ and four sp^2^ quaternary carbons (including three ester carbonyls). The NMR spectroscopic data of **2** (Figures S5–S7, and Table 1) showed the presence of 1,1-disubstituted double bond (δ_C_ 143.0 C, 116.3 CH_2_; δ_H_ 5.23 s, 2H). Three ester carbonyls (δ_C_ 172.2, 171.0 and 170.4) were assigned from the ^13^C NMR spectrum and their signals were correlated with the methylene protons (δ_H_ 2.30, 2H, m) of an *n*-butyrate and protons of two acetate methyl (δ_H_ 2.08 s and 2.07 s, each 3H), respectively, indicated the presence of one *n*-butyrate and two acetoxy groups. The remaining three degrees of unsaturation identified **2** also a tricyclic diterpenoid. The molecular framework of this compound was also established by COSY and HMBC correlations (Figure 1). Comparison of the NMR data of **2** with those of the known compound krempfielin E (**4**) [16] revealed that **2** is the C-12 acetylated derivative of krempfielin E. The stereochemistry of compound **2** was determined by the NOESY correlations as shown in Figure 2.

Many cytotoxic and anti-inflammatory eunicellin-based compounds have been discovered from soft corals [4,5,6,7,8,9,10,11,12,13,14,15,16,17,18,19,20,21,22,23,24,25,26,27]. Recently, we isolated several eunicellins with anti-inflammatory activity by significantly inhibiting superoxide anion generation and elastase release in human neutrophiles induced by FMLP/CB [17,18]. The same *in vitro* anti-inflammatory effects of the diterpenoids **1** and **2** also were tested. At a concentration of 10 μM, compound **2** exhibited some anti-inflammatory activity in reducing the generation of superoxide anion (13.17% ± 2.09% inhibition) and in inhibiting the elastase release (11.09% ± 5.55% inhibition), relative to the control cells stimulated with FMLP/CB only (Table 2). The cytotoxicity of **1** and **2** against five human carcinoma cell lines, CCRF-CEM, K562, Molt 4, T47D and DLD-1 were also evaluated by the MTT assay, and both compounds did not show activity against the proliferation of these cancer cell lines. The impurity of compound **2** might affect the bioactivity and the biological activities of **3** were not measured due to the paucity of this compound.

**Table 2:** Effects of compounds **1** and **2** on superoxide anion generation and elastase release in FMLP/CB-induced human neutrophils.

Compound	Superoxide Anion		Elastase	
	Inh %	IC_50_ (μM) ^a^	Inh %	IC_50_ (μM)
**1**	5.46 ± 5.19	>10	2.99 ± 2.82	>10
**2**	13.17 ± 2.09 **	>10	11.09 ± 5.55	>10

Percentage of inhibition (Inh %) at 10 μM concentration. Results are presented as mean ± S.E.M. (the standard error of mean) (*n* = 3 or 4). ** *p* < 0.01 compared with the control value; and ^a^ Concentration necessary for 50% inhibition (IC_50_).

## 3. Experimental Section

### 3.1. General Experimental Procedures

Optical rotations were measured on a JASCO P-1020 polarimeter (Jasco, Tokyo, Japan). IR spectra were recorded on a JASCO FT/IR-4100 spectrophotometer (Jasco). ESIMS were obtained with a Bruker APEX II mass spectrometer (Bruker Daltonics, Billerica, MA, USA). The NMR spectra were recorded in CDCl_3_, either on a Varian UNITY INOVA-500 FT-NMR (Varian Inc., Palo Alto, CA, USA) or a Varian 400MR FT-NMR (Varian Inc.). Silica gel (Merck, Darmstadt, Germany, 230–400 mesh) was used for column chromatography. Precoated silica gel plates (Kieselgel 60 F-254, 0.2 mm, Merck) were used for TLC analysis (Merck). High-performance liquid chromatography (HPLC) was performed on a Hitachi L-2130 HPLC apparatus (Hitachi, Tokyo, Japan) equipped with Hitachi L-2455 diode array detector (Hitachi) and a Supelco C18 column (250 mm × 21.2 mm, 5 μm, Supelco, Bellefonte, PA, USA).

### 3.2. Animal Material

*C. krempfi* was collected by hand using scuba off the coast of Penghu islands of Taiwan in June 2008, at a depth of 5–10 m, and stored in a freezer until extraction. A voucher sample (specimen No. 200806CK) was deposited at the Department of Marine Biotechnology and Resources, National Sun Yat-sen University, Kaohsiung, Taiwan*.*

### 3.3. Extraction and Separation

The octocoral (1.1 kg fresh wet weight) was collected and freeze-dried. The freeze-dried material was minced and extracted exhaustively with EtOH (3 × 10 L). The EtOH extract of the frozen organism was partitioned between CH_2_Cl_2_ and H_2_O. The CH_2_Cl_2_-soluble portion (14.4 g) was subjected to column chromatography on silica gel and eluted with EtOAc in *n*-hexane (0%–100% of EtOAc, stepwise) and then further with MeOH in EtOAc with increasing polarity to yield 41 fractions. Fraction 30, eluted with MeOH–EtOAc (1:10), was rechromatographed over a silica gel open column using acetone in *n*-hexane (0%–100% of acetone, stepwise) as the mobile phase to afford six subfractions (A1–A6). Subfraction A4 (eluted with *n*-hexane–acetone 1:1) was further separated by reverse phase HPLC (CH_3_CN–H_2_O, 1.5:1) to afford compound **3** (1.0 mg). Fraction 32, eluted with MeOH–EtOAc (1:10), was rechromatoraphed over a silica gel open column using acetone in *n*-hexane (0%–100% of acetone, stepwise) as the mobile phase to afford eight subfractions (B1–B8). Subfraction B2 (eluted with *n*-hexane–acetone 2:1) was separated by reverse phase HPLC (CH_3_CN–H_2_O, 1:1) to afford compound **1** (2.2 mg). Subfraction B3 (eluted with *n*-hexane–acetone 1.8:1) was subjected to reverse phase HPLC (CH_3_CN–H_2_O, 1:1.6) and yielded compound **2** (2.0 mg).

#### 3.3.1. Krempfielin Q (**1**)

Colorless oil; 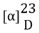 = +84.7 (*c* 0.77, CHCl_3_); IR (neat) *v_max_* 3445, 3076, 2963, 2932, 1733, 1646, 1455, 1373, 1237, 1183, and 1067 cm^−1^; ^13^C and ^1^H NMR data, see Table 1; ESIMS *m*/*z* 505 [M + Na]^+^; HRESIMS *m*/*z* 505.2779 [M + Na]^+^ (calcd. for C_2__6_H_42_O_8_Na, 505.2777).

#### 3.3.2. Krempfielin R (**2**)

White powder; 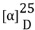 = +82.0 (*c* 0.57, CHCl_3_); IR (neat) *v_max_* 3444, 3038, 2963, 2930, 1732, 1650, 1456, 1373, 1239, 1182 and 1074 cm^−1^; ^13^C and ^1^H NMR data, see Table 1; ESIMS *m*/*z* 563 [M + Na]^+^; HRESIMS *m*/*z* 563.2835 [M + Na]^+^ (calcd. for C_2__8_H_44_O_10_Na, 563.2832).

### 3.4. Cytotoxicity Testing

Cell lines were purchased from the American Type Culture Collection (ATCC). Cytotoxicity assays of compounds **1** and **2** were performed using the MTT (3-(4,5-dimethylthiazol-2-yl)-2,5-diphenyl-tetra-zolium bromide) colorimetric method [28,29].

### 3.5. In Vitro Anti–Inflammatory Assay–Superoxide Anion Generation and Elastase Release by Human Neutrophils

Human neutrophils were obtained by means of dextran sedimentation and Ficoll centrifugation. Measurements of superoxide anion generation and elastase release were carried out according to previously described procedures [30,31]. LY294002, a phosphatidylinositol-3-kinase inhibitior, was used as a positive control for inhibition of superoxide anion generation and elastase release with IC_50_ values of 1.88 ± 0.45 and 4.12 ± 0.92 μM, respectively. Briefly, superoxide anion production was assayed by monitoring the superoxide dismutase-inhibitable reduction of ferricytochrome c. Elastase release experiments were performed using MeO-Suc-Ala-Ala-Pro-Val-*p*-nitroanilide as the elastase substrate [32].

## 4. Conclusions

Two new eunicellin-based diterpenoids **1** and **2** were isolated together with a known one from the continuing investigation of a soft coral *Cladiella krempfi.* Although both compounds were not cytotoxic towards a limited panel of cancer cell lines, **2** could inhibit the generation of superoxide anion and the release of elastase in FMLP/CB-induced human neutrophils.

## Supplementary Materials

Supplementary figures can be found at http://www.mdpi.com/1422-0067/15/12/21865/s1.
